# Persistent and Alarming Hæmorrhage from the Bladder

**Published:** 1885-08

**Authors:** H. C. Ghent

**Affiliations:** Ex-Pres’t Texas State Med. Ass’n., Belton, Texas


### PERSISTENT AND ALARMING HÆMORRHAGE FROM THE BLADDER.

*By H. C. Ghent, M. D., Ex-Pres't Tex. Med. Ass'n, Belton Tex.*

For Daniel's Texas Medical Journal.

A GRAPHIC report of the following case may not be without some interest to the average medical reader and practitioner :

On the 23rd of May last, assisted by Drs. Frank Allen, W. N. Rogers and Taylor Hudson, I operated for vesico—vaginal fistula. The patient, Miss D. P., age 17 years, was brought to me from an adjoining county, during the spring of 1884, for examination and, perhaps, for treatment. I learned from the mother and the family physician the following brief history: During the summer of 1883 she had an attack of something similar to purpura hœmorrhagica, accompanied, or rather followed, by a protracted fever of supposed malarial origin. During the time there was extensive sloughing of the vagina walls, and some weeks after recovery, it was discovered that there was complete occlusion of a portion of the vaginal tract. In attempting to relieve this condition, by a surgical operation, in order that the pent up menstural blood might escape, the consulting surgeon, accidently opened the bladder to a considerable extent, but succeeded in removing the depot of blood and relieving the agonizing pain. Upon examination, I found the vagina almost completely occluded, by a dense, cartilaginous, broad ring or band, situated about equal distance from the os uteri to the ostium vaginæ, being able to introduce only a number eight bougie. The fistula was situated just in front of the dense part of the stricture and a little to the right of the median line. I must confess that, considering my inexperience in this special field of practice, coupled with the extensive fibrinous bands almost entirely closing the vagina, and the extensive solution of continuity found in the vesico—vaginal septum—I was reluctant to give much encouragement from the source relief had been asked. I advised the parents of this angelic creature to carry her to Emmet, or Bozeman,or Thomas,or Marcy,or Battey, or Campbell or some other distinguished gynecologist; but the reply was, "Doctor, we are not able financially, and we are willing to risk you in the case." I replied, "I will do, then, the best I can."

I adopted Bozeman's plan for dividing the adventitious tissues, using his knife and his sponge dilators. At three different times, three preliminary operations for dividing the bands were performed, before undertaking to close the fistula. In the second preliminary operation, there occurred an alarming hemorrhage, some eight or ten hours after dividing a portion of the stricture, *there being no hemorrhage at the time.*

The operation for closing the fistula was unattended by hemorrhage or any unusual occurrence or trouble ; but it was seemingly impossible to bring the patient under the *surgical* influence of ether, as two hours were consumed in the inhalation of Squibb's solution of ether, through Allis' inhaler, and only a semi-conscious state induced. I may state that the same signal failure attended our efforts on two former occasions in the same subject. But at each time a few inhalations of chloroform soon induced complete anæsthesia.

For the first forty-eight hours after the operation, there was slight hemorrhage from the bladder, but none from the vaginal surface of wound. Nothing remarkable occurred from the hour of the operation, 2 o'clock p. m., the 23rd, until 10.30 p. m., the 27th. No constitutional disturbance, except a slight elevation of temperature about noon of the day last mentioned. In my absence to the country, Dr. Allen was called at the hour indicated, in consequence of a frightful hemorrhage from the bladder, attended by severe vesical tenesmus. I arrived a few minutes after,and found our patient very much excited and suffering excruciating paroxysmal pain, as clots of blood were forced from the bladder by muscular contraction. To relieve the agony, we at once injected into the cellular tissue of the arm Sul. Morph. gr. ⅓ and Sul. Atropia gr. 1-96. Also gave quinine grs. 5, by mouth. Washed out bladder with warm water, and left Dr. Allen, about midnight, with patient for balance of the night. At five o'clock a. m., 28th, was summoned, in great haste, in consequence of increased vesical hemorrhage. Found her suffering great pain from vesical tenesmus. Gave sul. Morph. gr. and Sul. Atropia gr. 1-96, by hypodermic needle, and immediately, by same process, Squibb's Fl. Ex. Ergot, M. x l. In thirty minutes, administered, per orem, Fl. Ex. Ergot ʒi. We now attempted to wash out bladder with warm water, (just as warm as the patient would tolerate) but found much difficulty in doing so, for the reason of the rapid formation and expulsion of blood coagula. From 6 p. m., to 11 p. m., used frequent intra-vaginal injections and frequently introduced catheter for the purpose of relieving distended bladder. At 9 a. m., was forced to repeat morphine and atrophia by hypodermic syringe.

At 3.30 p. m., fearful increase of hemorrhage, large coagula being frequently passed, but, at this time, without the previous degree of pain. At 3.45 p. m., the hemorrhage continuing with but slight, if any, remissions, washed out bladder with strong solution of tannic acid. At 4 p. m., gave, by the mouth, Gallic acid gr. 5.

At 4.30 p. m., used tannic acid dissolved in ice water. At 5.30 p. m., turned patient in Sims' left latteral position and examined, by means of his speculum, the vaginal aspect of the wound. Found all the stitches in place, the lips in perfect apposition and the wound presenting every appearance of healing by the first intention. No leakage, and I may remark, enpassant, there never was a drop of urine, or blood, that could be *demonstrated*, escaped from the bladder into the vagina, through the closed fistula, from the time the silver wires were twisted till the termination of the case.

At 6.30 p. m., passed small coagula and about one ounce of urine, considerably tinged with blood. No pain and much less hemorrhage since the last injection of tannin.

At 7 p. m., gave grs. 2 crude opium. Pulse 108, temperature about normal; the former much smaller in volume and greatly reduced in force as might be supposed. At 8 p. m., gave gallic acid gr. 5. Large clots now passing at intervals of from ten to thirty minutes. The suffering was so intense, at times, we were compelled to increase the amount of opium. The strong solutions of tannin in ice water doing no good, we concluded to try alum water, but this, too, proved a complete failure. Our patient becoming so much exsanguined and exhausted, we were forced to give brandy, and brandy and sweet milk frequently. We had now tried hot and cold water injections, simple and medicated, in the bladder; cold and hot water in the vagina; ice over the hypogastric, perineal and sacral regions; gallic acid and ergot by the stomach; ergot by hypodermic needle, but all to no purpose. We had a solution of Per. Sul. Ferri, in readiness, but hesitated, for reasons well known to the reader. Up to this time, no injection had given pain.

I had no idea that even a female urethra, of any age, was susceptible of sufficient dilatation as to allow such *enormous* masses of firmly clotted blood to pass unbroken. In order that a catheter might be introduced at all, without using violence, it had to be done *immediately* after the passage of a coagulum, as a delay of a *few moments* rendered its introduction seemingly impracticable, if not impossible.

It was now reaching 2 o'clock a. m., the 20th. My friend, Dr. Allen, had fallen into the arms of "tired nature's sweet restorer, balmy sleep," having been up with the patient all the previous night. No sound disturbed the awful stillness and solemnity of the hour, save an occasional deep, though suppressed sigh, from the grief-stricken mother, or a death-like moan from the expiring maiden. What an hour! What a responsibility! A precious life suspended upon a few frail threads and these being rapidly broken, one after another! What more shall be done? What more can be done to arrest the further escape of the vital fluid and thereby avert impending death? Thoughts were not *just now* crowding the mind, for this process had been going on for hours. I thought of removing the stitches, and, according to the suggestion of Dr. Emmet, "pushing a portion of a thin handkerchief through the fistula, then, as the ends are held," packing "a sufficient quantity of cotton into the bag thus formed," to make "a mass in shape like a door-knob, which presses against the bleeding surface when traction is made on the portion of the handkerchief outside of the fistula." I thought of attempting to pass, according to the suggestion of the same distinguished author, a suture "from the vagina, through the septum into the bladder, on the finger as a guide, then across to same distance on the other side, and out into the vagina again. In this way, the bleeding vessel, which comes from the neck of the urethra, or from the neck of the bladder, is ligated, as it were, in the fold of tissue, and the bleeding is arrested.

But, if either procedure had been practicable at that hour of the night, I do not believe there was sufficient physical strength remaining to have kept soul and body together during the operation.

I thought of Prof. R. A. F. Penrose's article on post partum hemorrhage and its treatment, published in *Gaillard's Medical Journal* in 1878 or '79, in which he strongly recommended apple vinegar in such cases, and, "thinks I to myself," if acetic acid or vinegar will exercise a controling influence over that frightful accident or complication in the lying-in chamber, why not over vesical hemorrhage? I knew that vinegar had been used as a styptic or hemostatic locally applied, for a time, on the other side of which the memory of the oldest doctor fails to reach, but I had no knowledge of it ever having been used for the arrest of hemorrhage from this organ. In arresting post partum hemorrhage it does *more* than causing the muscular tissue of the uterus to contract; it causes, by its presence, blood to clot over the mouths of the bleeding orifices, and also excites to contraction, the muscular walls of the bleeding vessels. Before risking the injection of the iron solution as a dernier resort, I concluded to try what virtue there might be in vinegar. Accordingly, I mixed with Oss of apple vinegar an equal quantity of ice water and had all things in readiness for passing the mixture through the bladder, *just after* the expulsion of a huge coagula.

This took place about 2 a. m., 29th. I at once introduced a soft, double-barreled catheter, (Tiemann's patent) and began the process of running the mixture slowly through the bladder. The contact of the vinegar with the interior of the bladder gave *very great pain*, my patient exclaiming in a faint tone, "Doctor, you are killing me." I persisted, and when I had irrigated the bladder with about twelve ounces of the mixture, the hemorrhage ceased never more to return from that minute until the present time. The young lady is now at home perfectly sound, so far as the bladder or the fistula in the vesico vaginal septum is concerned. Nothing, in a practice of thirty years, ever acted more *magically*.

Being anxious to know whether vinegar had ever been used in the treatment of this accident, I addressed a note to a number of distinguished gynecologists in this country, asking *that* as well as one or two other questions, and among the answers received up to this time, is the following from Dr. T. A. Emmet:

89 Madison Avenue, N. Y., June 23, 1885.

Dr. H. C. Ghent:

Belton, Texas.

*Dear Doctor:*—"In all the cases I have known of with hemorrhage, after closing a vesico vaginal fistula, the bleeding has been on the bladder side, and the blood has been passed with the urine. I do not know exactly what injections Dr. Peaslea used, but believe they were with the Per Sul. Iron largely diluted in water. I have never heard of vinegar being used for the purpose of arresting the loss of blood in the bladder."

Dr. Henry F. Campbell, of Georgia, writes me: "I am glad you did not inject Per Sul. Iron, as it would have made a clot as hard as a *brick bat*, which would have become a nucleus of a stone in the bladder and have given you a world of trouble."

I may state, in closing this hastily written report, that at some future time a complete history of this case will be given to the profession.

